# Dam inactivation in *Neisseria meningitidis*: prevalence among diverse hyperinvasive lineages

**DOI:** 10.1186/1471-2180-4-34

**Published:** 2004-08-31

**Authors:** Keith A Jolley, Li Sun, E Richard Moxon, Martin CJ Maiden

**Affiliations:** 1 The Peter Medawar Building for Pathogen Research and Department of Zoology, University of Oxford, South Parks Road, Oxford, OX1 3SY, UK; 2 Molecular Infectious Diseases Group, University of Oxford Department of Paediatrics, Weatherall Institute of Molecular Medicine, John Radcliffe Hospital, Oxford, OX3 9DS, UK

## Abstract

**Background:**

DNA adenine methyltransferase (Dam) activity is absent in many, but not all, disease isolates of *Neisseria meningitidis*, as a consequence of the insertion of a restriction endonuclease-encoding gene, the '*dam *replacing gene' (*drg*) at the *dam *locus. Here, we report the results of a survey to assess the prevalence of *drg *in a globally representative panel of disease-associated meningococci.

**Results:**

Of the known meningococcal hyper-invasive lineages investigated, *drg *was absent in all representatives of the ST-8 and ST-11 clonal complexes tested, but uniformly present in the representatives of the other hyper-invasive lineages present in the isolate collection (the ST-1, ST-4, ST-5, ST-32 and ST-41/44 clonal complexes). The patterns of sequence diversity observed in *drg *were consistent with acquisition of this gene from a source organism with a different G+C content, at some time prior to the emergence of present-day meningococcal clonal complexes, followed by spread through the meningococcal population by horizontal genetic exchange. During this spread a number of alleles have arisen by mutation and intragenic recombination.

**Conclusion:**

These findings are consistent with the idea that possession of the *drg *gene may contribute to the divergence observed among meningococcal clonal complexes, but does not have a direct mechanistic involvement in virulence.

## Background

*Neisseria meningitidis*, the causative agent of meningococcal meningitis and septicaemia, is a common inhabitant of the human nasopharynx, being asymptomatically carried by approximately 10% of the population [1,2]. There is evidence for extensive horizontal genetic exchange in populations of this antigenically and genetically variable Gram-negative bacterium [3-7] but, despite the diversity of carried meningococci, only a limited number of genotypes – the hyper-invasive lineages – are responsible for most reported disease [8]. These lineages have been identified by the techniques of multilocus enzyme electrophoresis [9] and multilocus sequence typing (MLST) [10] as clonal complexes. In recent years, the ST-1 complex (formerly subgroup I), ST-4 complex (subgroup IV), and ST-5 complex (subgroup III) have dominated meningococcal disease in Africa and Asia, while members of the ST-11 (ET-37) complex, ST-8 complex (cluster A4), ST-41/44 complex (lineage 3), and ST-32 (ET-5) complex have caused most disease in other parts of the world. Meningococci occasionally cause epidemic outbreaks of varying scale up to global pandemics, although at a given point in time disease in a given geographical locale is often dominated by a limited number of clonal complexes [8].

DNA adenine methyltransferase (Dam) is an enzyme involved in the mismatch repair system of bacteria. During DNA replication, only the parental strand is fully methylated by the enzyme, since methylation is not immediate, allowing a mismatch on the newly synthesised strand to be excised and replaced [11]. Disruption of Dam activity suggests that mismatch repair will be less effective, potentially allowing the creation of frameshift mutations in the homopolymeric or simple repeat motifs within the promoter or coding regions of surface antigen genes leading to the reversible switching of their expression state. This is an important mechanism found at bacterial loci that encode gene products that are advantageous under certain conditions but not others; such genes have been termed contingency loci [12]. There is extensive evidence for such loci in the meningococcal genome [13]. In particular, the ability to vary the expression state of surface-exposed cellular components is important for within host adaptation, for example by allowing evasion of the immune response in the nasopharynx. These mechanisms are also important in the occasional transition from harmless colonisation to invasive disease [14-16]. Examples of such loci described in the meningococcus to date include: the capsular polysaccharide [17]; the Opc outer membrane protein [18-20]; pili [21]; the PorA protein [22]; and opacity proteins [19,23].

Dam methylation has been implicated in modifying the virulence of a number of bacterial pathogens [24-26], but its role in *N. meningitidis *has been contentious. Bucci *et al*., 1999 [27] suggested that the absence of Dam activity was responsible for high rates of phase variation in the *siaD *capsule gene, resulting in an increased virulence of some strains. In addition, the latter work suggested that all pathogenic isolates lacked Dam activity, the *dam *gene inactivated by the insertion of a putative restriction endonuclease, named '*dam *replacing gene' (*drg*), with the genotypes *dam*^+^/*drg*^- ^and *dam*^-^/*drg*^+ ^being mutually exclusive. However, two subsequent studies [28,29] have found no effect. The *drg *gene encodes a restriction enzyme, *Nme*BII that is similar to the *Streptococcus pneumoniae Dpn*I restriction endonuclease which cleaves at GmeATC, but not at GATC, sequences [30,31].

In the present study the prevalence of *drg *and its association with disease-associated isolates of *N. meningitidis *was examined by a survey of its distribution in a collection of isolates chosen to represent the known diversity of pathogenic meningococci [10]. The results suggest that, while *drg *is present in some lineages but not others, this gene has spread in the meningococcal population by horizontal genetic exchange, possibly after the introduction of the *drg *gene from an exogenous source.

## Results

### Distribution of *dam *and *drg *among invasive meningococci

In all cases, there was an exact correlation with the presence of *dam *and the absence of *drg*, and vice-versa. Of the 84 isolates tested, 23 were indicated to be *dam*^+^/*drg*^- ^and 61 to be *dam*^-^/*drg*^+ ^by restriction analysis and PCR amplification (see Additional file 1). The presence of *dam *or *drg *was associated with particular clonal complexes. Of the hyper-invasive lineages included in the analysis, all ST-8 complex and ST-11 complex isolates were *dam*^+^/*drg*^-^, while all of the representatives of the ST-1, ST-4, ST-5, ST-41/44, and ST-32 complexes were *dam*^-^/*drg*^+^.

### Diversity of *drg *gene fragment sequences

**Figure 1:**
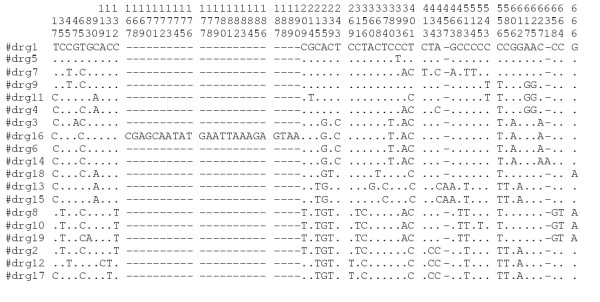
Variable sites in *drg *gene fragments

The level of nucleotide diversity, as represented by number of alleles and polymorphic sites, along with the p-distances seen within *drg *gene fragments was similar to that seen with the housekeeping genes used for MLST (Table 1), with a total of 19 different fragment sequences (*drg-1 *– *drg-19*) present in this collection. The ratio of non-synonymous to synonymous substitutions (*d*_*N*_/*d*_*S*_, 0.11) was also similar to that seen in the MLST gene fragments. Three *drg *gene fragments (*drg-13*, *drg-14 *and *drg-15*) exhibited polymorphisms resulting in single base frameshifts, two resulting in stop codons (Figure 1) and one fragment (*drg-16*) had an insertion corresponding to a 24-base direct repeat. The G+C content of *drg *at 42.4% was lower than that of the housekeeping genes, which were in the range 51.1–57.4%.

**Figure 2:**
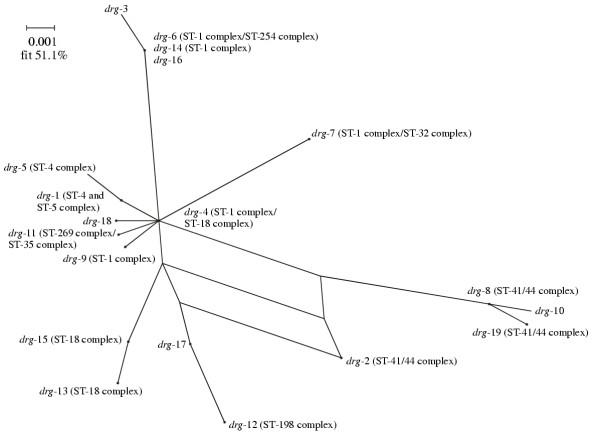
**Splits graph of *drg *gene fragments. **The *drg *alleles found in the major *drg*^+ ^hyper-invasive lineages are annotated.

Split decomposition analysis of the *drg *fragment sequences generated a star phylogeny with some net-like phylogenetic structure involving the *drg-2 *allele (Figure 2). The distribution of the different *drg *sequences amongst the clonal complexes is also shown in Figure 2.

## Discussion

The meningococcus is an example of an accidental pathogen, a commensal organism that rarely causes disease and which gains no evolutionary benefit from this process [32]. A complete understanding of the diseases caused by such organisms is dependent upon an appreciation of the mechanisms by which harmless carriage develops into invasive disease [33]. The observation that some meningococcal genotypes, the hyper-invasive lineages, are more likely to cause disease than others [8,10] suggests that comparative studies of disease-associated and carried meningococci may identify genetic factors responsible for disease [34]. In this context, the suggestion that the possession of a single genetic change, the insertion of the *drg *gene at the *dam *locus, was essential for virulence in meningococci [27] was attractive as it provided a single characteristic associated with the disease phenotype. Further, this proposal provided a plausible mechanistic explanation, namely the promotion of rapid switching of expression of contingency genes, for example those encoding the capsule which represents the best defined meningococcal virulence determinant [35]. This is consistent with the evidence that inactivation of Dam has been shown to affect rates of recombination and phase change in other species [25,36].

The data presented here, however, do not support the contention that the *drg *insertion is preferentially associated with disease-associated meningococcal isolates, even those expressing serogroup B capsular polysaccharide. There was no evidence of a difference between disease-associated and carried meningococcal isolates with 76% (45/59) of disease-associated isolates and 75% (11/15) of carrier isolates possessing the *drg *insertion. The isolate collection employed by the present investigation was chosen to be globally representative of meningococcal disease in the latter part of the 20^th ^century, with multiple examples of each of the major hyper-invasive meningococci identified over this period. When analysed by clonal complex, which identifies hyper-invasive lineages, it was apparent that the *drg *insertion was absent from all of the isolates representing the ST-8 and ST-11 clonal complexes, both major hyper-invasive lineages but, conversely, *drg *was present in all representatives of the hyper invasive lineages represented by the ST-1, ST-4, ST-5, ST-41/44, and ST-32 complexes. This association was independent of the serogroup expressed by the isolates and provided a likely explanation of the earlier observations [27]. As meningococcal disease in a given locale at a given time tends to be dominated by a limited number of clonal complexes [8], it is likely from the results provided here that a given sample of disease-associated isolates collected from a given locale will be uniform at the *dam *locus. At a given point in time they may be dominated by *drg *containing meningococci, while at other times isolates without this insertion might dominate. For example, as both the ST-1 and ST 32 complexes contain the *drg *insertion, this insertion would be prevalent among the disease isolates recovered by the pandemic outbreaks caused by these clonal complexes [37,38]; conversely, the spread of ST-11 meningococci [39,40] would result in an increased prevalence of disease-associated meningococci without the *drg *insertion. This observation highlights the importance of assembling genetically defined isolate collections with a prescribed sampling frame if comparisons of meningococci with potential for virulence are to be undertaken.

It has been suggested that the *drg *gene has been introduced into *Neisseria *from an exogenous source [30]. The difference in the G+C% content of the *drg *gene compared to the meningococcal housekeeping genes was consistent with this idea; however, the levels of diversity among the *drg *alleles, which was similar to that observed in the meningococcal housekeeping genes, along with the reported occurrence of *drg *in other *Neisseria *species [30], suggested that this was a relatively old event predating the emergence of present-day clonal complexes. The ratio of non-synonymous to synonymous substitutions (*d*_N_/*d*_S_), which was also similar to that observed in meningococcal housekeeping genes, suggested that the *drg *gene had been subject to stabilising selection for conservation of function since its putative introduction into the meningococcal population, although the possession of frame shift mutations in some of the *drg *alleles demonstrated that, as with *N. lactamica*, not all meningococci possessing the *drg *insertion expressed the endonuclease [30]. In other respects the distribution of the gene among clonal complexes and the patterns of nucleotide sequence variation observed were similar to those seen in meningococcal housekeeping genes, suggesting that it is subject to similar selection pressures [41].

The presence of multiple methylation systems is a characteristic of the *Neisseria *and may be related to the transformable nature of these organisms [42]. Whole genome comparisons of meningococci belonging to different clonal complexes frequently yield different restriction modification systems as the principal detectable genetic differences [43,44]. It is attractive to speculate that the genetic isolation of clonal complexes may be promoted by such differences [45] and in this respect the insertion of *drg *in the *dam *locus, replacing a methylase with an endonuclease, may provide a potent barrier to genetic exchange from *dam*^+ ^to *drg*^+ ^meningococci; indeed, while there was evidence for the occasional horizontal genetic exchange of *drg *alleles among the genetically diverse meningococci that possess the insertion, there was no evidence for the transformational loss of *drg *from a given clonal complex among the isolates examined here. This concept of genetic isolation among lineages receives some support from the possession of *porB2 *alleles by members of the *dam*^+ ^ST-8 and ST-11 complexes and *porB3 *alleles by the clonal complexes with the *drg *insertion [9,39], but the evidence for limitations in gene flow between these subgroups of meningococci is not at present conclusive.

## Conclusions

While it is possible that the possession of the *drg *insertion may influence meningococcal population structure, the data presented here do not support a direct association of this genotype with meningococcal virulence.

## Methods

### Bacterial isolates

The 84 isolates used in this study (see Additional file 1) were a subset of a collection of 107 diverse meningococci assembled to develop and validate the MLST scheme for *Neisseria meningitidis *[10]. The collection included representatives of the major hyper-invasive lineages and has been characterised at many genetic loci (full details of this isolate collection are available at ).

### Restriction digestion of chromosomal DNA

The methylation status of the chromosomal DNA of each isolate was determined by restriction digestion with the restriction endonucleases *Dpn*I, *Dpn*II, and *Sau*3AI, followed by separation of the digestion products by agarose gel electrophoresis. Each enzyme specifically recognises a cognate target site of GATC and cleaves this site depending on whether it is methylated or not: *Dpn*I cleaves this target sequence only when N^6^-methyladenine is present within the recognition sequence; *Dpn*II cleaves only unmethylated sites, and *Sau*3AI cleaves both methylated and unmethylated target sequences.

### PCR amplification and nucleotide sequence determination

The presence of the *dam *gene was determined by polymerase chain reaction (PCR) performed using *dam *specific primers, DamF1 (5' – TAAAATGGGCAGGCGGCA – 3') and DamB2 (5' – CGTAAGGGGGATCGCAAT – 3'). These amplify a 534 bp fragment from the 5' end of the *dam *gene in *dam*+ strains but not from *dam*-strains. The presence of *drg *was determined by PCR using primers DrgF1 (5' – CATGAATTTATTTTTCGATA – 3') and DrgB2 (5' – AATTTGCAACTGTTGGCG – 3') that bind to *drg *internal sites and produce a 705 bp fragment in isolates containing the *drg *gene. PCR amplification was also performed using primer pairs Drg5F (5' – TGTCTAAAGAACTCAAAG – 3') / DrgB3 (5' – CGGTATCGAAAAATAAAT – 3') and Drg3F (5' – ATCCATCCAATTTCCCCA – 3') / DamB5 (5' – AAATGCCGTCTGAA – 3') based on *dam *and *drg *coding regions in order to confirm that dam inactivation was due to *drg *insertion.

Amplicons corresponding to the *drg *gene were purified by precipitation with poylethylene glycol and sodium chloride as described previously [46] and their nucleotide sequences determined on both strands by cycle sequencing. BigDye™ terminators (ABI, Foster city, California) and the same primers as used for amplification were employed in the extension reactions and the labelled reaction products separated on an ABI 3700 automated DNA sequencer. Each fragment was sequenced at least once on each strand and the sequences assembled with the STADEN suite of computer programs. Each unique *drg *sequence was assigned an arbitrary allele number in order of discovery.

### Data analysis

Percentage G+C was calculated using the program START [47]. Split decomposition analysis of the *drg *gene fragments was performed using SPLITSTREE, version 3.1 [48]. The proportion of non-synonymous to synonymous substitutions was calculated by the method of Nei and Gojobori [49] using the program MEGA2 [50].

## Authors' contributions

KAJ carried out the nucleotide sequencing and analysis of the data. LS performed the PCR screening of the isolates. ERM and MCJM conceived of the study and participated in its design and coordination.

## Supplementary Material

Additional File 1 Bacterial isolates used in the study Click here for file

## Figures and Tables

**Table 1:** Genetic variation in MLST and *drg *loci

Locus	No. of alleles (no./100 isolates)	No. (%) of polymorphic sites	dN/dS	Average p-distance	%G+C
*abcZ*	15 (14)	75 (17.4)	0.05	0.052	51.1
*adk*	10 (9.4)	17 (3.7)	0.02	0.012	52.3
*aroE*	18 (16.8)	166 (34)	0.293	0.106	55.6
*fumC*	19 (17.8)	38 (8.2)	0.024	0.022	57.4
*gdh*	16 (15)	28 (5.6)	0.05	0.019	52.3
*pdhC*	24 (22.4)	80 (16.7)	0.07	0.054	55.9
*pgm*	21 (19.6)	77 (17)	0.121	0.050	54.2
*drg*	19 (31.1*)	48 (7.4) †	0.11	0.021	42.4

*only 61 isolates contained *drg*

† excluding indels – length of fragment: 650
